# History matters: in the past, present & future of the NLM

**DOI:** 10.5195/jmla.2021.1206

**Published:** 2021-04-01

**Authors:** Jeffrey S. Reznick, Kenneth M. Koyle

**Affiliations:** 1 Supervisory historians on the federal staff of the US National Library of Medicine (NLM) of the National Institutes of Health (NIH) where they serve, respectively, as Chief and Deputy Chief of the NLM History of Medicine Division. They completed this article as part of their official duties, with support from NLM

In February 2015, the director of the National Institutes of Health (NIH) charged an advisory committee with articulating a strategic vision for the National Library of Medicine (NLM) to ensure that it remains an international leader in biomedical and health information [1]. In its final report later that year, the committee recognized NLM as follows:

… a source of support for training the next generation of data scientists and librarians, the place to learn about the past and explore the history of medicine and biomedical research, a source of new knowledge and standards as a result of its intramural research programs, and a repository of data for use in myriad research studies. The remarkable work of NLM has generated international goodwill and reflected positively on NIH and the United States. In fact, for many, NLM is the most visible face of NIH [2].

With regard to NLM's role as a national library with a globally appreciated collection that spans ten centuries and originates from nearly every part of our world, the committee recognized that its “unparalleled collection of primary historical sources and growing digital collections of medical artifacts are depended upon by historical researchers and educators,” among a wide variety of stakeholders.

Based on these and other observations, the committee formulated a series of recommendations, including the following:

- NLM should strengthen its role in fostering the future generation of professionals in biomedical informatics, data science, library sciences, and related disciplines through sustained and focused training efforts.
- NLM should maintain, preserve, and make accessible the nation's historical efforts in advancing biomedical research and medicine, thereby ensuring that this legacy is both safe and accessible for long-term use [3].

During the five years since the release of the report, a period during which public feedback [4] helped inform the NLM strategic plan for 2017-2027 [5], history has continued to matter at NLM, with its History of Medicine Division achieving many collaborative contributions toward the advancement of the library in the 21st century—indeed, for the benefit of historical research today and tomorrow. While the list of these contributions is too long to detail here, a few salient examples include the following:

## 1. Leading and contributing to the NLM Web Collecting and Archiving Working Group.

Together, we continue to identify and select web and social media content documenting the Coronavirus disease (COVID-19) pandemic as part of NLM's Global Health Events web archive collection. Including more than 8,600 seed URLs totaling 2.4-plus terabytes of data as of June 2021, the collection includes federal, state, and local government COVID-19 pages, websites of disaster relief agencies and nongovernmental organizations (NGOs), and content documenting life in quarantine, prevention measures, vaccine development, the experiences of healthcare workers, patients, and more. The group continues to review recommended content for inclusion in this ever-growing archive, scoping and running crawls of content using Archive-It, the leading web archiving service for collecting and accessing cultural heritage on the web, and Conifer (formerly Webrecorder), the web archiving service that creates an interactive copy of an archived web page.

Moreover, the NLM Web Collecting and Archiving Group continues to engage with other cultural heritage organizations archiving the history of COVID-19, including a group spearheaded by the leadership of the Smithsonian National Museum of American History, as well as a group of federal agency representatives who meet regularly to discuss their respective collecting initiatives. The group also continues to engage regularly with the Society of American Archivists Web Archiving Section, the Archive-It community, and the National Digital Stewardship Alliance and is contributing to and following the growing list of institutions collecting COVID-19-related content maintained by Documenting the Now, a resource that is “responding to the public's use of social media for chronicling historically significant events as well as demand from scholars, students, and archivists, among others, seeking a user-friendly means of collecting and preserving this type of digital content” [6]. Nominations for content to include in NLM's Global Health Events collection remain welcome via nlmwebcollecting@nlm.nih.gov. NLM also continues to participate as an institutional contributor to a broader International Internet Preservation Consortium (IIPC) Novel Coronavirus outbreak web archive collection. Learn more about NLM's efforts in the recently published *Journal of the Medical Library Association* article, “The National Library of Medicine Global Health Events web archive, coronavirus disease (COVID-19) pandemic collecting,” [7] and about the broader context of documenting the pandemic as described in an article published in *Nature* on December 17, 2020, titled “What are COVID archivists keeping for tomorrow's historians?” [8].

## 2. Advancing the NLM-National Endowment for the Humanities (NEH) interagency partnership.

Over its eight-year history, this inter-agency partnership has brought together scholars, scientists, librarians, archivists, curators, technical information specialists, healthcare professionals, cultural heritage professionals, and others in the humanities and biomedical communities to share expertise and develop new research agendas at the intersection of biomedicine and the humanities. The recent extension of this partnership, to 2024, followed the April 2020 NLM-NEH virtual research symposium, Reporting, Recording, and Remembering the 1918 Influenza Epidemic, during which Virginia Tech students studying the history of data in social context presented their research on various aspects of the 1918 flu epidemic in the United States [9]. Other impactful NLM-NEH collaborations have included the following:

- Viral Networks: An Advanced Workshop in Digital Humanities and Medical History, held in January 2018, which yielded the peer-reviewed, open-access book *Viral Networks: Connecting Digital Humanities and Medical History*, published by VT Publishing [10];
- Images and Texts in Medical History: An Introduction to Methods, Tools, and Data from the Digital Humanities, a workshop held in April 2016 that explored emerging approaches to the analysis of texts and images in the field of medical history;
- Shared Horizons: Data, Biomedicine, and the Digital Humanities, a symposium convened in April 2013 that explored the intersection of digital humanities and biomedicine; and
- An Epidemiology of Information: New Methods for Interpreting Disease and Data, a symposium convened in October 2013 that explored new methods for large-scale data analysis of epidemic disease.

## 3. Revealing data in NLM's historical collections.

Through a series of curated posts on our popular blog [11], we have been reflecting on how NLM has long been a steward of vast collections of data, even as it now gains recognition as the home of data science at NIH. In this respect, this ongoing series explores what data-minded researchers from a variety of disciplines are learning from centuries of preserved data associated with a variety of topics—from 17th-century bills of mortality to the 1918 Spanish Flu, tuberculosis in 19th-century America, measles in the 20th century, patient privacy, and to NLM resources like GenBank and the Index-Catalogue of the Library of the Surgeon General's Office—and how data-focused study of these events can help us think about the future preservation and uses of the data we collect today.

## 4. Exploring new and innovative methods to care for NLM's historical collections through state-of-the-art research and practice.

Our conservators developed a technique called Photoshop® Assisted Spectroscopy, a way to digitally track color shift of ink under different conditions, to identify and implement the safest environment for the genetic code charts handwritten by the Nobel Prize-winning NIH scientist Marshall Nirenberg from data he and his team collected from experiments that determined how protein sequence was dictated by the sequence of precursor ribonucleic acids (RNAs) [12]. Currently, the conservation team is working with conservators and scientists from NLM, the Smithsonian Institution and Penn State University to examine historical methods of processing leather used in bookbinding to identify the causes of accelerated degradation in leather processed using modern methods. This analysis will be enhanced by using Fourier Transform Infrared Spectroscopy (FTIR), a technique that can reveal the molecular structure of material, to identify the differences in manufacturing practices and the causes of leather degradation. This work ensures that our collections will exist for generations of future researchers, and the new methods and processes developed here will help conservators around the world preserve other historical collections.

## 5. Acquiring historically important works from around the world.

Hand in hand with our prospective acquisition of historically significant born-digital material, we continue to acquire works retrospectively to enhance NLM's historical collections as a record of human experience of health and disease. Recently, we acquired the following:

- The papers of Dr. Louis W. Sullivan, former U.S. Secretary of Health and Human Services and dean and president of Morehouse School of Medicine, documenting his efforts to educate the public on the dangers of tobacco use, introduce new and improved food labels, initiate a $100 million minority male health and injury prevention initiative, and increase the National Institutes of Health budget by more than $5 billion;
- A set of more than 2,200 hospital postcards representing more than 1,300 U.S. hospitals during the first seven decades of the 20th century, the period when hospitals emerged to become the chief locus of care for persons who were sick, injured, or dying. Institutions pictured in this new collection range from small and little-known private hospitals in small towns to major academic medical centers in our largest cities;
- The first edition of the first book to describe the first officially sanctioned dissection in Japan: *On the Viscera* (1759) by Toyo Yamawaki, a prominent medical scientist and pioneer in experimental medicine who, through his work, contributed to the modernization of Japanese medicine; and
- The first edition of the foundational work that introduced quantitative experimentation into biological science: Santorio Santorio's 1614 text *Ars de statica medicina aphorismorum sectionibus septem comprehensa* [On Static Medicine]. Santorio's work joins a number of classic texts held by the NLM that reflect and document data-driven scientific discovery over the centuries, from multiple foreign-language first editions of Darwin's *Origin of Species* to Florence Nightingale's *Notes on Nursing* to bills of mortality documenting the Great Plague of the 17th century.

Interested researchers can learn more about our recent acquisitions by searching the “recent acquisitions” tag on our blog [13].

## 6. Curating new historical exhibitions and raising awareness of NLM's trustworthy biomedical and consumer health information.

Our award-winning exhibition program has a long history of surfacing important stories residing in the NLM's historical collections and sharing them with audiences across the United States and around the world through our popular traveling exhibitions and online adaptations. Among the newest exhibitions are the following:

- *Care and Custody: Past Responses to Mental Health*, curated by scholar Anne E. Parsons, PhD (University of North Carolina at Greensboro), which explores the treatment of people with mental health conditions throughout history, with specific attention to such treatment over the past 200 years in the United States and the tension that has existed between care and custody as responses to mental health issues [14]; and
- *This Lead is Killing Us: Citizens Fighting Lead Poisoning in Their Communities*, curated by scholar Richard M. Mizelle, Jr, PhD (University of Houston), which tells an important story of citizen action taken against an environmental danger—lead exposure—that can cause neurological problems and sometimes even death [15].

## CONCLUSION: THE FUTURE OF HISTORY AT THE NLM

**Figure 1:**
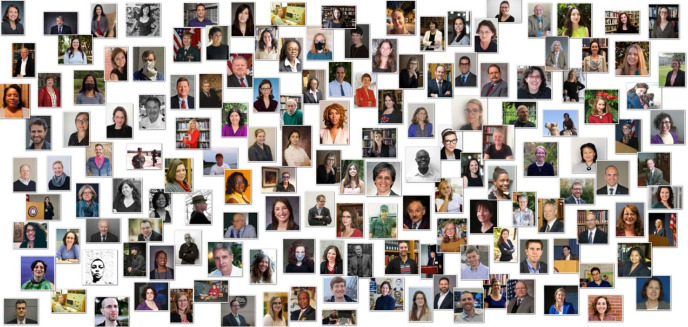
Contributors to Circulating Now—the popular NLM History of Medicine Division blog—who, collectively, have shared the myriad ways NLM historical collections and programs have informed research, teaching, and public service.

The future of history at the NLM appears bright, as it remains a key component of the library's mission, operations, and engagements with a wide range of stakeholders located in the United States and around the world. These stakeholders include not only historians of medicine but also individuals from a variety of disciplines who are advancing their research, teaching, and public service through engagement with our vast collections, dynamic exhibitions, and related resources: from historians of art, film, and photography to archivists, librarians, and museum professionals; from digital humanists to data scientists; from literary, legal, and women's studies scholars to medical and public health professionals; from journalists to public policy professionals. In fact, one could say that the individuals who regularly engage with us are as diverse as the NLM historical collections themselves. Figure 1 shows just some of our many patrons, along with many of our outstanding staff of archivists, curators, historians, and librarians, who have contributed to our popular blog, sharing the myriad ways our collections and related programs have informed research, teaching, and public service. Together, we represent the very future of history at the NLM as our institution—the world's largest biomedical library, located on the campus of the NIH—continues to document, preserve, and advance understanding of the past, present, and future human condition.

## References

[1] National Institutes of Health Advisory Committee to the Director. National Library of Medicine (NLM) Working Group Charge. Bethesda, MD: National Institutes of Health; 2 2 2015. 2 p. Available from: https://www.nih.gov/sites/default/files/about-nih/nih-director/statements/collins/ACD_NLM_WG_Charge_FINAL.pdf.

[2] National Institutes of Health Advisory Committee to the Director. National Library of Medicine (NLM) Working Group Final Report. Bethesda, MD: National Institutes of Health; 11 6 2015. 17 p. Available from: https://acd.od.nih.gov/documents/reports/Report-NLM-06112015-ACD.pdf.

[3] National Institutes of Health Advisory Committee to the Director. National Library of Medicine (NLM) Working Group Final Report. p. 10-11.

[4] National Institutes of Health Request for Information (RFI): Strategic Plan for the National Library of Medicine, National Institutes of Health [Internet] NLM. [8 11 2016]. Available from: https://grants.nih.gov/grants/guide/notice-files/not-lm-17-002.html.

[5] National Library of Medicine. NLM Launches 2017-2027 Strategic Plan: A Platform for Biomedical Discovery and Data-Powered Health [Internet] NIH. [5 3 2018]. Available from: https://www.nlm.nih.gov/news/NLM_Launches_2017_to_2027_Strategic_Plan.html.

[6] Documenting the Now. About [Internet]. Washington University in St. Louis, the University of California, Riverside, and the Maryland Institute for Technology in the Humanities. [cited 10 Feb 2021]. Available from: https://www.docnow.io/.

[7] Moffatt C, Speaker S. The National Library of Medicine Global Health Events web archive, coronavirus disease (COVID-19) pandemic collecting. J Med Libr Assoc. 2020 10; 108(4): 656-662. Available from: 10.5195/jmla.2020.1090. 33013228 PMC7524615

[8] Spinney L. What are COVID archivists keeping for tomorrow's historians? Nature. 2020 12; 588(7839): 578-583. https://media.nature.com/original/magazine-assets/d41586-020-03554-0/d41586-020-03554-0.pdf.

[9] Research Symposium: Reporting, Recording, and Remembering the 1918 Influenza Epidemic [videocast]. National Institutes of Health. Bethesda, MD; 2020 4 29. Available from https://videocast.nih.gov/watch=36347.

[10] Ewing ET, Randall K. (eds) (2019) Viral Networks: Connecting Digital Humanities and Medical History. Blacksburg: Virginia Tech Publishing. DOI: 10.21061/viral-networks.

[11] Circulating Now: From the Historical Collections of the National Library of Medicine [Internet]. Bethesda, MD: National Library of Medicine. History of Medicine Division. Revealing Data; [cited 10 Feb 2021]. Available from: https://circulatingnow.nlm.nih.gov/tag/data/.

[12] Circulating Now: From the Historical Collections of the National Library of Medicine. Preserving Nirenberg's Genetic Code Chart; [cited 10 Feb 2021]. Available from: https://circulatingnow.nlm.nih.gov/2014/09/16/preserving-nirenbergs-genetic-code-chart/.

[13] Circulating Now: From the Historical Collections of the National Library of Medicine. Recent Acquisitions; [cited 10 Feb 2021]. Available from: circulatingnow.nlm.nih.gov/tag/recent-acquisitions.

[14] Care and Custody: Past Responses to Mental Health [Internet]. Bethesda, MD: National Library of Medicine. History of Medicine Division. 24 9 2020 [cited 10 Feb 2021] Available from: https://www.nlm.nih.gov/exhibition/careandcustody/index.html.

[15] This Lead is Killing Us: A History of Citizens Fighting Lead in Their Communities [Internet]. Bethesda, MD: National Library of Medicine. History of Medicine Division. 15 10 2019 [cited 10 Feb 2021] Available from: https://www.nlm.nih.gov/exhibition/thisleadiskillingus/index.html.

